# Long-Standing Motor and Sensory Recovery following Acute Fibrin Sealant Based Neonatal Sciatic Nerve Repair

**DOI:** 10.1155/2016/9028126

**Published:** 2016-06-29

**Authors:** Natalia Perussi Biscola, Luciana Politti Cartarozzi, Rui Seabra Ferreira Junior, Benedito Barraviera, Alexandre Leite Rodrigues de Oliveira

**Affiliations:** ^1^Department of Tropical Diseases, Botucatu Medical School, São Paulo State University (UNESP), 18618-000 Botucatu, SP, Brazil; ^2^Center for the Study of Venoms and Venomous Animals (CEVAP), São Paulo State University (UNESP), 18610-307 Botucatu, SP, Brazil; ^3^Department of Structural and Functional Biology, Institute of Biology, University of Campinas, 13083-970 Campinas, SP, Brazil

## Abstract

Brachial plexus lesion results in loss of motor and sensory function, being more harmful in the neonate. Therefore, this study evaluated neuroprotection and regeneration after neonatal peripheral nerve coaptation with fibrin sealant. Thus, P2 neonatal Lewis rats were divided into three groups: AX: sciatic nerve axotomy (SNA) without treatment; AX+FS: SNA followed by end-to-end coaptation with fibrin sealant derived from snake venom; AX+CFS: SNA followed by end-to-end coaptation with commercial fibrin sealant. Results were analyzed 4, 8, and 12 weeks after lesion. Astrogliosis, microglial reaction, and synapse preservation were evaluated by immunohistochemistry. Neuronal survival, axonal regeneration, and ultrastructural changes at ventral spinal cord were also investigated. Sensory-motor recovery was behaviorally studied. Coaptation preserved synaptic covering on lesioned motoneurons and led to neuronal survival. Reactive gliosis and microglial reaction decreased in the same groups (AX+FS, AX+CFS) at 4 weeks. Regarding axonal regeneration, coaptation allowed recovery of greater number of myelinated fibers, with improved morphometric parameters. Preservation of inhibitory synaptic terminals was accompanied by significant improvement in the motor as well as in the nociceptive recovery. Overall, the present data suggest that acute repair of neonatal peripheral nerves with fibrin sealant results in neuroprotection and regeneration of motor and sensory axons.

## 1. Introduction

Upper and lower limb innervation is greatly affected by brachial and lumbosacral plexus lesion, leading to loss of motor and sensory function [1–7]. In the neonate, such injuries are even more harmful, since surgical treatment is limited and neuronal loss is particularly enhanced by neurotrophic factors deprivation. Nonexistent or limited handling of neonatal proximal lesions results in limb dysfunction and neuropathic pain [8–12].

Experimentally, a well-accepted model to mimic axotomy injury retrograde repercussion to spinal neurons is the neonatal peripheral nerve axotomy [13–18]. Sciatic nerve transection, early after birth, results in significant degeneration of spinal motoneurons as well as sensory neurons present in the dorsal root ganglia. Also, a number of interneurons, present in deep spinal cord laminae, degenerate due to the loss of input and/or output. Importantly, exogenous treatment with neurotrophins transiently rescues a significant number of neurons, leading to the possibility of axonal regrowth and regeneration [19, 20].

To date, neonatal peripheral nerve repair following neurotmesis is largely limited due to technical drawbacks. In fact, to perform end-to-end coaptation by epineural/perineural suturing in the newborn represents a major challenge. On the contrary, reconnection of transected stumps by using biocompatible sealants may significantly facilitate the process, as well as taking the advantage of employing the adhesive as scaffold to engraft stem cells, as well as neurotrophic substances, to the injury site.

Fibrin sealant has been applied in neurosurgery for decades, being effective and biocompatible, with no side effects to the nervous system microenvironment [21]. It is made of two main components: fibrinogen and thrombin. Together, when activated, their interaction mimics the coagulation cascade, forming a stable and adhesive clot [22]. Currently, the source of commercial fibrin sealant is the human blood. In this regard, much effort by the Center for the Study of Venoms and Venomous Animals (CEVAP) has been made to produce a new fibrin sealant that uses a serine protease from a Brazilian snake (*Crotalus durissus terrificus*) with thrombin-like activity. Also, in order to avoid human blood, bubaline-derived blood serum cryoprecipitate has been used as the fibrinogen source. Among several advantages, the nonhuman fibrin glue hampers transmission of hepatitis and HIV/AIDS [23–27]. Also, it has a customizable formulation that can be adjusted to increase or decrease fibrin clot formation and stability.

Although anatomical repair of spinal roots and other lesioned plexus components constitute the primary approach, additional strategies are necessary to enhance neuroprotection and to improve the regenerative response of severed neurons. This may be achieved by stem cell therapy that has shown particularly important positive effects following nervous system injury [28–37]. In this regard, we have already shown evidence that mononuclear cell therapy produced neuroprotection, preservation of synapses in the spinal cord, and reduction of glial reaction following dorsal and ventral horn adult lesion [38, 39].

The present work investigated the viability and efficiency of neonatal sciatic nerve end-to-end coaptation, performed with the application of fibrin sealant. We show that both commercial and customized nonhuman fibrin adhesives perform equally well and allow motor and sensory recovery up to 12 weeks after injury.

## 2. Material and Methods

### 2.1. Fibrin Sealant

Nerve stumps coaptation was performed by using either a commercially available fibrin sealant-Tissucol (Baxter®, Vienna, Austria) or a patented fibrin sealant derived from snake venom, kindly supplied by the Center for the Study of Venoms and Venomous Animals (CEVAP) of UNESP (patent registration numbers BR1020140114327 and BR1020140114360). At the time of use, the components were previously thawed, reconstituted, mixed, and applied [24–27, 31].

### 2.2. Animals and Surgery Procedure

Two-day-old (P2) neonatal Lewis rats were anesthetized by hypothermia and subjected to unilateral sciatic nerve transection at mid-thigh level. Animals were divided into three groups: axotomy alone (AX, *n* = 30), in which a 2 mm segment of the proximal stump was resected in order to assure absence of contact between stumps; axotomy followed by end-to-end CEVAP's fibrin sealant coaptation (AX+FS, *n* = 30); axotomy followed by end-to-end commercial fibrin sealant coaptation (AX+CFS, *n* = 30). An overview of the surgical procedure is illustrated in supplementary Figure S1 (in Supplementary Material available online at http://dx.doi.org/10.1155/2016/9028126). All experimental conditions were assessed 4, 8, and 12 weeks after lesion. An additional nonoperated control group was specifically utilized for the motor test evaluation (*n* = 5). Also, in order to evaluate the retrograde degeneration of motoneurons following nerve transection without proximal stump resection, an additional axotomy control group was evaluated following stump reapposition (AX-SR; *n* = 5).

Both fibrin sealants used herein are composed of three separate solutions: (1) fibrinogen, (2) calcium chloride, and (3) thrombin or thrombin-like (in the case of the serine protease derived from snake venom). During surgical repair, the first two components were applied and the proximal and distal stumps were approximated in an end-to-end fashion. The third sealant component was then added for polymerization. The sciatic nerve was gently shifted to assure the stability of the coaptation and to evaluate the success of the repair.

The Institutional Committee for Ethics in Animal (CEUA/UNICAMP) and The Center of Experimental and Ethical Animal (CEEA/UNESP) approved all experiments (proc. number 2593-1 and 904-2011, resp.), which were performed in accordance with the guidelines of the NIH and the Brazilian College for Animal Experimentation. The animals were housed using a 12 h light/dark cycle and controlled temperature (23°C), with free access to food and water. Pups were maintained with the mother until 21 days old, being weaned afterwards.

### 2.3. Specimen Preparation

Animals were anaesthetized with an overdose of Kensol (Xylasine, Köning, Argentina, 10 mg/kg) and Vetaset (Ketamine, Fort Dodge, USA, 50 mg/kg), and the vascular system was rinsed by transcardial perfusion with phosphate buffer 0.1 M (pH 7.4). For neuronal survival counting and the immunohistochemical evaluation, subjects (*n* = 5 for each group) were fixed by vascular perfusion of 10% formaldehyde in phosphate buffer (pH 7.4). The lumbar intumescence was dissected out, postfixed overnight, washed in phosphate buffer, and stored in sucrose 10, 20, and 30% for 24 h each, before freezing. Transverse cryostat sections (12 *μ*m thick) of the lesioned spinal cord segments (L4, L5, and L6) were obtained, transferred to gelatin coated slides, and stored at −22°C until use. For sciatic nerve regeneration analysis, the rats were transcardially perfused with 200 mL of a fixative containing 2.5% glutaraldehyde and 1.0% paraformaldehyde in 0.1 M phosphate buffer (pH 7.4). The sciatic nerve ipsi and contralateral to the lesion were removed and stored overnight in the same fixative at 4°C. The specimens were then osmicated, dehydrated, and embedded in Durcupan (Fluka ACS, Steinheim, Switzerland). The resin blocks were trimmed and semithin sections (0.5 *μ*m) were obtained and stained with 0.7% Sudan Black in 70% of ethanol solution for morphometric analysis.

### 2.4. Neuronal Survival

Counting of motoneurons survival was performed on spinal cord sections (*n* = 5 for each group, ipsi and contralateral ventral horn, 4, 8, and 12 weeks after lesion). Cryostat transverse sections were stained for 3–5 min in aqueous 1% cresyl fast violet solution at room temperature. The sections were then washed in distilled water, dehydrated, and mounted with Entellan (Merck, Darmstadt, Germany).

The motoneurons were identified based on their morphology and location in the ventral horn (dorsolateral lamina IX). Only cells with visible nucleus were counted for every twentieth section (totalizing an interval of 240 *μ*m between sections) along the lumbar enlargement, both on the ipsilateral and contralateral sides of each spinal cord.

The percentage of surviving cells was analyzed by the ratio of absolute numbers of motoneurons, counted per section, on the lesioned and nonlesioned sides, respectively, and multiplying the result by 100. Abercrombie's formula [40] was used to correct the duplicate counting of neurons: *N* = *nt*/(*t* + *d*), where *N* is the corrected number of neurons counted, *n* is the number of cells counted, *t* is the thickness of the sections (12 *μ*m), and *d* is the average diameter of neuronal nuclei. Since the difference in size significantly affects cell counts, the value of *d* was calculated specifically for each experimental group (ipsilateral and contralateral). In this sense, the nuclear diameter of 15 randomly picked neurons from each group was measured with IMAGEJ software (version 1.33, National Institutes of Health, USA) and the mean value was calculated.

### 2.5. Immunohistochemistry

Slides containing transverse spinal cord sections were incubated for 45 min in a 3% BSA solution followed by incubation with the following primary antibodies: mouse anti-synaptophysin (Dako, Glostrup, Denmark 1 : 200) rabbit anti-GFAP (Abcam, 1 : 1500), and rabbit anti-Iba1 (Waco, 1 : 700). The primary antibodies were diluted in a solution containing 1% BSA (bovine serum albumin) and 2% triton in PB 0.1 M (phosphate buffer). All sections were incubated for three hours in a moist chamber at room temperature. After rinsing in PB, the sections were incubated with a Cy3-conjugated secondary antiserum (1 : 250, Jackson Immunoresearch, West Grove, PA, USA) for 45 min in a moist chamber at room temperature. The sections were then rinsed in PB, mounted in a mixture of glycerol/PB (3 : 1), and observed with a Leica DM5500B microscope coupled with a Leica DFC345 FX camera.

For quantitative measurements, three representative images of the ipsilateral and contralateral ventral horn were captured from each animal for all experimental groups (*n* = 5 for each group, ipsi and contralateral ventral horn, 4, 8, and 12 weeks after lesion). For analysis of synaptophysin immunolabeling, the integrated density of pixels was measured in eight areas surrounding each lateral motor nucleus motoneuron, in the anterior horn of the spinal cord. For the analysis of anti-glial fibrillary acidic protein (GFAP) and anti-ionized calcium binding adaptor molecule 1 (Iba1) antibodies, the integrated density of pixels was measured in the lateral region of the spinal cord ventral horn, as described by Oliveira et al. and Freria et al. (2010, Supporting Information) [20, 41]. The integrated density of pixels was calculated for each section and then as a mean value for each spinal cord. The data are represented as the mean ± standard error (SE) for each group.

### 2.6. Sciatic Nerve Regeneration

Morphometry, regenerated axon area, and counting analyses were performed by sampling at least 30% of each nerve cross-section (magnification of 1,000x) using a bright field microscope (*n* = 5 for each group, ipsi and contralateral ventral horn, 4, 8, and 12 weeks after lesion). Sampling bias was avoided by spreading the micrographs systematically over the entire cross-section, according to the procedure proposed by Mayhew and Sharma [42]. These images were used for counting the total number of myelinated fibers. For each specimen, two fields were used to measure the perimeter and diameter of myelinated fibers and the myelin area, using Adobe Photoshop CS6 Extended. These values were used to calculate the myelinated axon diameter, fiber diameter, myelin thickness (fiber diameter, axon diameter/2), and “*g*” ratio (axon diameter/fiber diameter) that indicate the relative success of the regeneration process as well as myelination process. The data are shown as mean ± SE.

### 2.7. Electron Microscopy

Lumbar spinal cords (*n* = 3 for each group, ipsi and contralateral ventral horn, 4 weeks after lesion) were dissected out and stored overnight in fixative at 4°C. The specimens were then trimmed, osmicated, dehydrated, and embedded in Durcupan (Fluka). Ultrathin sections from the L4–L6 segments were collected on formvar coated copper grids, counterstained with 2% uranyl acetate and lead citrate, and examined under a transmission electron microscope operating at 120 KV (Tecnai Biotwin G2 Spirit, FEI Company, The Netherlands). Neurons with large cell bodies (~35 *μ*m in diameter), found in the sciatic motoneuron pool and cut in the nuclear plane, were identified as *α*-motoneurons by the presence of C-type nerve terminals and chromatolysis (axotomized side). The cell surfaces were then sequentially digitalized at a magnification of 11,000x with an Eagle 2K video camera (FEI Company, The Netherlands) connected to a computer system, and the images mounted together using vector graphics software. Synaptic terminals opposing the motoneuron somata were identified and their numbers per 100 *μ*m of cell membrane, as well as the membrane covering of all the terminals (calculated in percentage of membrane length), were determined using the measurement tool of Image J software (version 1.33u, National Institutes of Health (NIH), USA).

The terminals were typed as F-type (flattened synaptic vesicles or flattened and spherical vesicles that contain glycine/gamma-aminobutyric acid (GABA) as neurotransmitter), S-type (spherical synaptic vesicles that contain glutamate as the neurotransmitter), or C-type (presence of a subsynaptic cistern and acetylcholine as the neurotransmitter), according to the procedure described by Conradi in 1969 [43] (Supplementary Figure S2). The distance between consecutive nerve terminals covering the motoneurons was also determined. A total of 24 sciatic *α*-motoneurons from three animals per group (two neurons per animal in four groups, AX, AX+FS, AX+CFS, and contralateral) were quantified. The data are represented as the mean ± SE.

### 2.8. Functional Analysis

#### 2.8.1. Motor Recovery

Motor function was analyzed using the peroneal functional index (PFI) by the walking track test (CatWalk system, Noldus Inc., The Netherlands; *n* = 5 for each group). In this system, the animal crosses a walkway with an illuminated glass floor. A high speed video camera Gevicam (GP-3360, USA) equipped with a wide-angle lens (8.5 mm, Fujicon Corp., China) is positioned underneath the walkway and the paw prints are automatically recorded and classified by the software. The paw prints from each animal were obtained twice a week from the third week of life. PFI was calculated as the distance between the third toe and hind limb pads (print length) and the distance between the first and fifth toes (print width). Measurements of these parameters were obtained from the right (unlesioned) and left (lesioned) paw prints, and the values were calculated using the following formula described by Bain and colleagues in 1989 [44]:

(1) PFI=174.9×EPL−NPLNPL+80.3×ETS−NTSNTS−13.4,

where N: normal or nonoperated side; E: experimental or operated; PL: print length; TS: total toe spread, or distance between first to fifth toes.

#### 2.8.2. Nociceptive Sensory Recovery

The electronic von Frey test (Insight Instruments Inc., Ribeirão Preto, SP, Brazil) was utilized to evaluate sensory function recovery (*n* = 5 for each group) [45]. For that, the rats were kept inside acrylic boxes for twenty minutes before the experiment for habituation. Nociception threshold was measured at the plantar region by touching the skin with a pipette tip adapted to a force transducer, which is connected to a digital counter that shows the force, in grams, used to trigger the paw flinch. Sensibility intensity was evaluated twice a week, from the fourth week after surgery. Hyperalgesia was measured by stimulating the paw surface for six times, and the results were averaged and shown as a mean ± SE. In order to avoid foot pad lesion and inflammation, the maximum stimulation weight (cut-off threshold) was set to 70 g. Each data collection was carried out in duplicate and in a blinded manner.

### 2.9. Statistical Analysis

The data are presented as mean ± SE, and the differences between groups were considered significant when the *P* value was <0.05 (*∗*), <0.01 (*∗∗*), and <0.001 (*∗∗∗*). Statistical analysis was performed with GraphPad Prism 4.0 software. In this sense, data were subjected to ANOVA followed by Bonferroni post hoc test for parametric data or Mann-Whitney *U* test for nonparametric data.

## 3. Results

### 3.1. Nerve Coaptation at Neonatal Stage Increases Long Term Neuronal Survival

**Figure 1:**
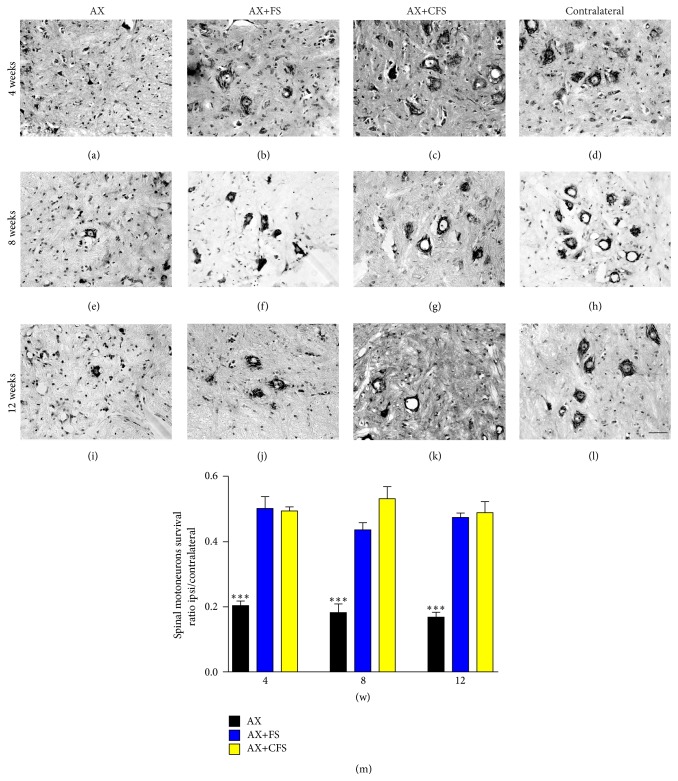
Nissl-stained spinal cord transverse sections at lamina IX illustrating the neuroprotective effect on motoneurons, 4, 8, and 12 weeks following P2 sciatic nerve transection and repair. Note the decreased number of motoneurons ipsilateral to the lesion and the improvement of neuronal survival in the coaptation groups. (a, b, c) Ipsilateral side, 4 weeks after lesion, groups AX, AX+FS, and AX+CFS, respectively. (d) Contralateral side, 4 weeks after lesion. (e, f, g) Ipsilateral side, 8 weeks after lesion, groups AX, AX+FS, and AX+CFS, respectively. (h) Contralateral side, 8 weeks after lesion. (i, j, k) Ipsilateral side, 12 weeks after lesion, groups AX, AX+FS, and AX+CFS, respectively. (l) Contralateral side, 12 weeks after lesion. Scale bar = 50 *μ*m. (m) Ratio of neuronal survival 4, 8, and 12 weeks following P2 sciatic nerve transection and repair. Both coaptation groups displayed a significantly increased neuronal survival in all survival times analyzed. Mean ± SE. AX: axotomy; AX+FS: axotomy followed by coaptation with fibrin sealant derived from snake venom; AX+CFS: axotomy followed by coaptation with commercial fibrin sealant.

Neuronal survival was investigated as the ipsi/contralateral ratio of motoneurons present at ventral horn lamina IX. After 4, 8, and 12 weeks, a severe degeneration motoneuron was observed in the AX group. Coaptation groups performed equally well and presented statistically significant rescue of axotomized motoneurons (Figure 1, Table S1). No significant differences between the numbers of motoneurons on the contralateral side in the different experimental conditions were observed. Additionally, axotomy alone with immediate reapposition of the stumps and followed by 2 mm proximal stump resection did not differ with regard to motoneuron survival (Supplementary Figure S3).

### 3.2. Preservation of Synapses by Fibrin Sealant Coaptation

**Figure 2:**
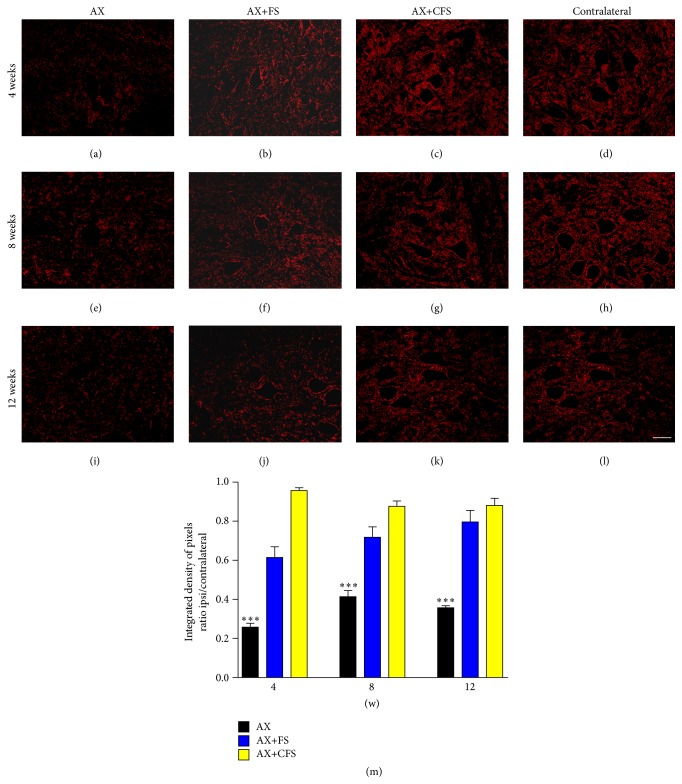
Immunohistochemical analysis of the spinal cord ventral horn stained with antisynaptophysin, 4, 8, and 12 weeks, following P2 sciatic nerve transection and repair. A significant preservation of synaptophysin immunoreactivity is observed in both coaptation groups in all time analyzed. (a, b, c) Ipsilateral side, 4 weeks after lesion, groups AX, AX+FS, and AX+CFS, respectively. (d) Contralateral side, 4 weeks after lesion. (e, f, g) Ipsilateral side, 8 weeks after lesion, groups AX, AX+FS, and AX+CFS, respectively. (h) Contralateral side, 8 weeks after lesion. (i, j, k) Ipsilateral side, 12 weeks after lesion, groups AX, AX+FS, and AX+CFS, respectively. (l) Contralateral side, 12 weeks after lesion. Scale bar = 50 *μ*m. (m) Synaptic covering 4, 8, and 12 weeks after injury, obtained by the ratio IL/CL (ipsi/contralateral sides) of the integrated density of pixels at lamina IX. Observe the significant reduction of synaptic elimination in both groups repaired with fibrin sealant, in all survival times analyzed. Mean ± SE. AX: axotomy; AX+FS: axotomy followed by coaptation with fibrin sealant derived from snake venom; AX+CFS: axotomy followed by coaptation with commercial fibrin sealant.

Quantitative measurements of synaptophysin immunoreactivity in the sciatic motor nuclei after axotomy (AX) and after axotomy followed by coaptation (AX+FS; AX+CFS) were carried out 4, 8, and 12 weeks after injury. Axotomy group showed significant reduction of immunoreactivity ipsilateral to the lesion side. On the contrary, synaptic covering was preserved following coaptation in all survival times analyzed (Figure 2, Supplementary data Table S2).

### 3.3. Early Postnatal Nerve Coaptation Resulted in Downregulation of Astroglial and Microglial Reaction

**Figure 3:**
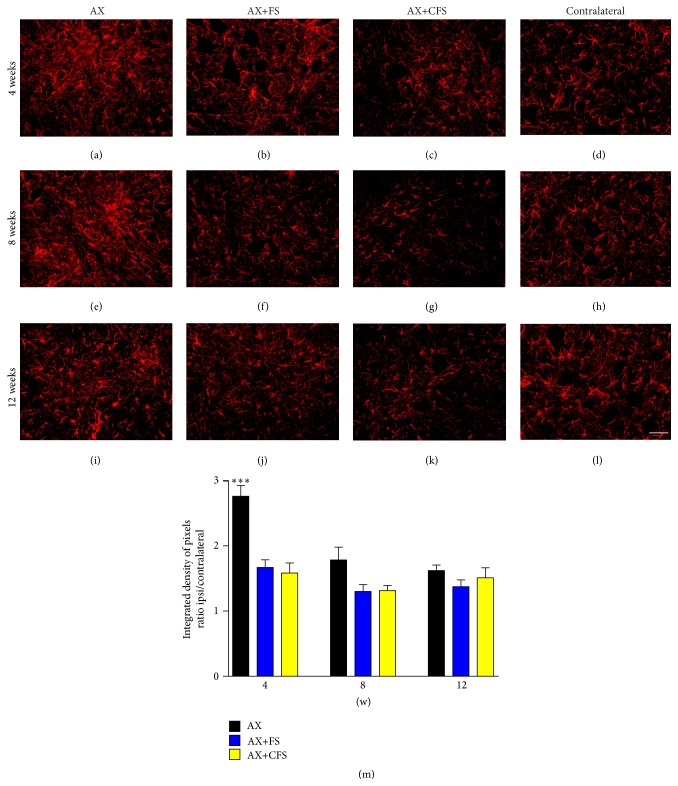
Immunohistochemical analysis of the spinal cord ventral horn stained with glial fibrillary acid protein (GFAP), 4, 8, and 12 weeks, following P2 sciatic nerve transection and repair. A decrease in astrogliosis is observed in both coaptation groups, 4 weeks after lesion. (a, b, c) Ipsilateral side, 4 weeks after lesion, groups AX, AX+FS, and AX+CFS, respectively. (d) Contralateral side, 4 weeks after lesion. (e, f, g) Ipsilateral side, 8 weeks after lesion, groups AX, AX+FS, and AX+CFS, respectively. (h) Contralateral side, 8 weeks after lesion. (i, j, k) Ipsilateral side, 12 weeks after lesion, groups AX, AX+FS, and AX+CFS, respectively. (l) Contralateral side, 12 weeks after lesion. Scale bar = 50 *μ*m. (m) The mean ratio, obtained by the ratio IL/CL (ipsi/contralateral sides) of the integrated density of pixels at lamina IX. Observe the significant reduction of astrogliosis in both groups repaired with fibrin sealant, 4 weeks after lesion. Mean ± SE. AX: axotomy; AX+FS: axotomy followed by coaptation with fibrin sealant derived from snake venom; AX+CFS: axotomy followed by coaptation with commercial fibrin sealant.

**Figure 4:**
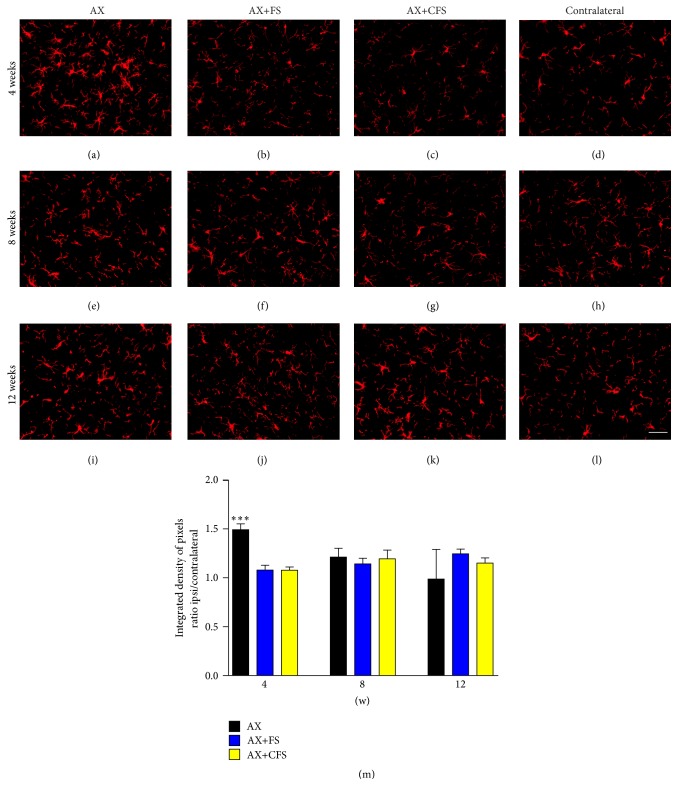
Immunohistochemical analysis of the spinal cord ventral horn stained with ionized calcium binding adaptor protein (Iba1), 4, 8, and 12 weeks, following P2 sciatic nerve transection and repair. A decrease in microglial reaction is observed in both coaptation groups, 4 weeks after lesion. (a, b, c) Ipsilateral side, 4 weeks after lesion, groups AX, AX+FS, and AX+CFS, respectively. (d) Contralateral side, 4 weeks after lesion. (e, f, g) Ipsilateral side, 8 weeks after lesion, groups AX, AX+FS, and AX+CFS, respectively. (h) Contralateral side, 8 weeks after lesion. (i, j, k) Ipsilateral side, 12 weeks after lesion, groups AX, AX+FS, and AX+CFS, respectively. (l) Contralateral side, 12 weeks after lesion. Scale bar = 50 *μ*m. (m) The mean ratio, obtained by the ratio IL/CL (ipsi/contralateral sides) of the integrated density of pixels at lamina IX. Observe the significant reduction of microglial reaction in both groups repaired with fibrin sealant, 4 weeks after lesion. Mean ± SE. AX: axotomy; AX+FS: axotomy followed by coaptation with fibrin sealant derived from snake venom; AX+CFS: axotomy followed by coaptation with commercial fibrin sealant.

A significant increase in astrocyte reactivity after axotomy was observed four weeks after lesion (Figure 3, Supplementary data Table S3). The coaptation with fibrin sealant decreased such astrogliosis in all time points analyzed. Similar results were observed in microglial reactivity, where groups with coaptation showed decreased microglial reaction, particularly at four weeks after lesion (Figure 4, Supplementary data Table S4).

## 4. Nerve Coaptation Allowed Myelinated Axons Recovery 

### 4.1. Nerve Area and Number of Regenerated Axons

Although absence of spontaneous regeneration following axotomy alone was observed, acute coaptation (AX+FS, AX+CFS) resulted in axonal growth with sensorimotor recovery. Area measurements showed no significant difference between AX+FS and AX+CFS groups, although such nerves presented reduced dimensions as compared to the contralateral (nonlesioned) samples (Table 1).

**Figure 5:**
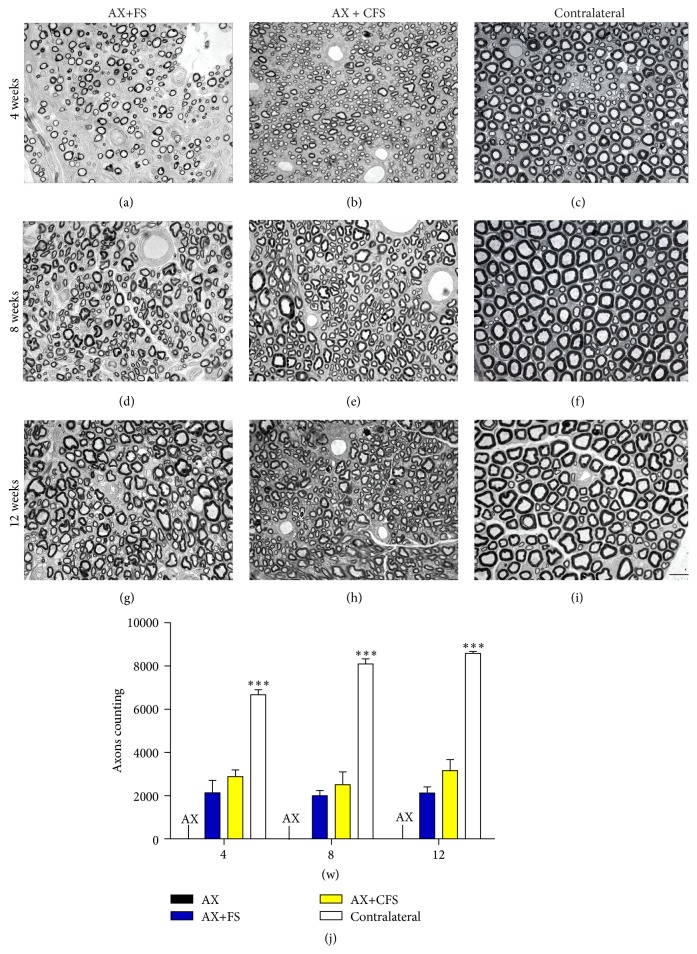
Representative micrographs of estimated number of regenerated myelinated axons of the sciatic nerve, 4, 8, and 12 weeks, following P2 sciatic nerve transection and repair. Note smaller fibers and counting in both coaptation groups compared with the contralateral nerve. Axotomy only group did not show any regenerated fiber. (a, b) Ipsilateral side, 4 weeks after lesion, groups AX+FS and AX+CFS, respectively. (c) Contralateral side, 4 weeks after lesion. (d, e) Ipsilateral side, 8 weeks after lesion, groups AX+FS and AX+CFS, respectively. (f) Contralateral side, 8 weeks after lesion. (g, h) Ipsilateral side, 12 weeks after lesion, groups AX+FS and AX+CFS, respectively. (i) Contralateral side, 12 weeks after lesion. Scale bar = 10 *μ*m. (j) Estimated number of regenerated myelinated fibers following P2 sciatic nerve transection and repair. Mean ± SE. AX: axotomy; AX+FS: axotomy followed by nerve coaptation with fibrin sealant derived from snake venom; AX+CFS: axotomy followed by coaptation with commercial fibrin sealant.

Additionally, the estimation of total number of axons revealed no significant differences between coaptation groups (Figure 5, Supplementary data Table S5), although significantly reduced in comparison to the normal nerve.

### 4.2. Morphometric Analysis of Regenerated Nerves

**Figure 6:**
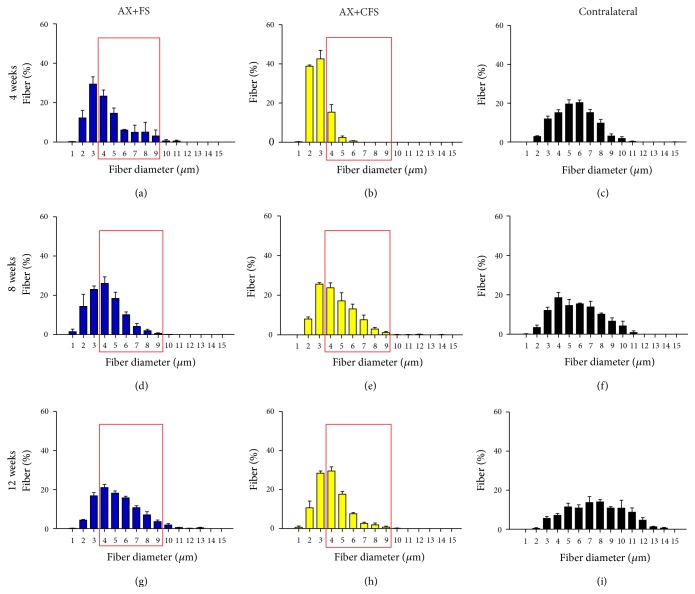
Frequency distribution of fiber diameter of regenerated fibers, 4, 8, and 12 weeks, following P2 sciatic nerve transection and repair. (a, b) Ipsilateral side, 4 weeks after lesion, groups AX+FS and AX+CFS, respectively. (c) Contralateral side, 4 weeks after lesion. (d, e) Ipsilateral side, 8 weeks after lesion, groups AX+FS and AX+CFS, respectively. (f) Contralateral side, 8 weeks after lesion. (g, h) Ipsilateral side, 12 weeks after lesion, groups AX+FS and AX+CFS, respectively. (i) Contralateral side, 12 weeks after lesion. Note smaller diameter fibers in the group AX+CFS, 4 weeks after lesion. Red boxes highlight frequency intervals with greater differences among groups. AX: axotomy; AX+FS: axotomy followed by coaptation with fibrin sealant derived from snake venom; AX+CFS: axotomy followed by coaptation with commercial fibrin sealant.

**Figure 7:**
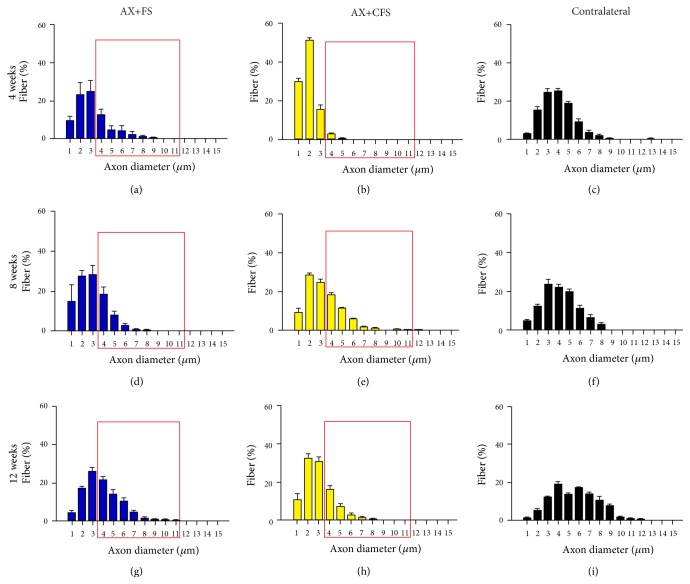
Frequency distribution of axon diameter of regenerated fibers, 4, 8, and 12 weeks, following P2 sciatic nerve transection and repair. (a, b) Ipsilateral side, 4 weeks after lesion, groups AX+FS and AX+CFS, respectively. (c) Contralateral side, 4 weeks after lesion. (d, e) Ipsilateral side, 8 weeks after lesion, groups AX+FS and AX+CFS, respectively. (f) Contralateral side, 8 weeks after lesion. (g, h) Ipsilateral side, 12 weeks after lesion, groups AX+FS and AX+CFS, respectively. (i) Contralateral side, 12 weeks after lesion. Note smaller axon diameters in the group AX+CFS, 4 weeks after lesion. Red boxes highlight frequency intervals with greater differences among groups. (AX) axotomy; (AX+FS) axotomy followed by coaptation with fibrin sealant derived from snake venom; (AX+CFS) axotomy followed by coaptation with commercial fibrin sealant.

**Figure 8:**
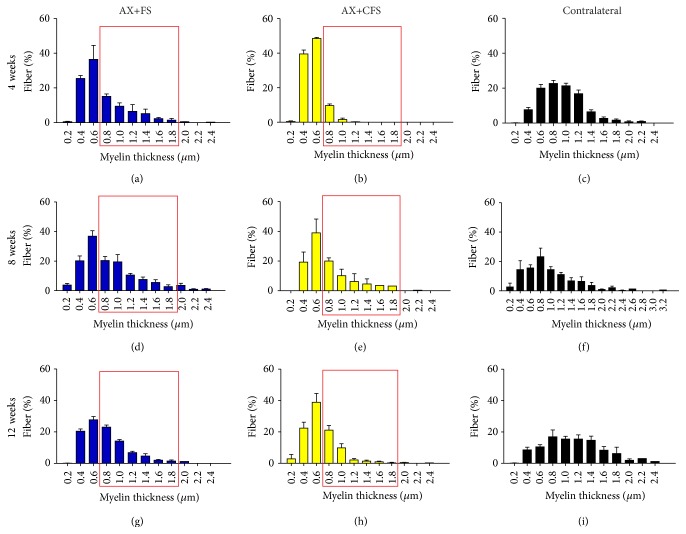
Frequency distribution of myelin thickness of regenerated fibers, 4, 8, and 12 weeks, following P2 sciatic nerve transection and repair. (a, b) Ipsilateral side, 4 weeks after lesion, groups AX+FS and AX+CFS, respectively. (c) Contralateral side, 4 weeks after lesion. (d, e) Ipsilateral side, 8 weeks after lesion, groups AX+FS and AX+CFS, respectively. (f) Contralateral side, 8 weeks after lesion. (g, h) Ipsilateral side, 12 weeks after lesion, groups AX+FS and AX+CFS, respectively. (i) Contralateral side, 12 weeks after lesion. Note decreased myelin thickness in the group AX+CFS, 4 weeks after lesion. Red boxes highlight frequency intervals with greater differences among groups. AX: axotomy; AX+FS: axotomy followed by coaptation with fibrin sealant derived from snake venom; AX+CFS: axotomy followed by coaptation with commercial fibrin sealant.

Morphometry revealed close to normal fiber distribution frequency following fibrin sealant from CEVAP coaptation. Of note, commercial fibrin adhesive displayed the worst values for all parameters analyzed at four weeks after lesion, indicating slower axonal regeneration progress (Figures 6, 7, and 8). Such initial difference was not present eight and twelve weeks after lesion, so that both coaptation groups performed in a similar fashion at later stages.

**Figure 9:**
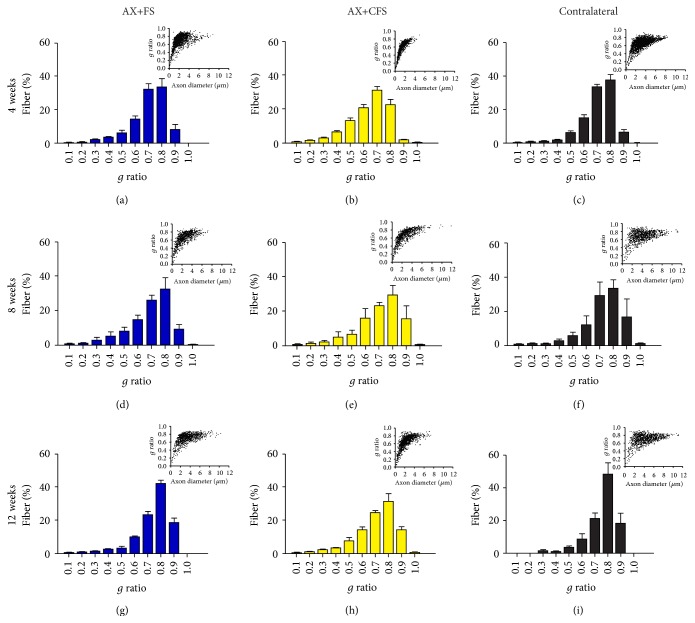
Frequency distribution of *g* ratio of regenerated fibers and dot plot of g ratio/axon diameter, 4, 8, and 12 weeks, following P2 sciatic nerve transection and repair. (a, b) Ipsilateral side, 4 weeks after lesion, groups AX+FS and AX+CFS, respectively. (c) Contralateral side, 4 weeks after lesion. (d, e) Ipsilateral side, 8 weeks after lesion, groups AX+FS and AX+CFS, respectively. (f) Contralateral side, 8 weeks after lesion. (g, h) Ipsilateral side, 12 weeks after lesion, groups AX+FS and AX+CFS, respectively. (i) Contralateral side, 12 weeks after lesion. Observe the shift toward the presence of greater axons with close to normal myelin thickness in both coaptation groups in all survival times analyzed. (AX) axotomy; AX+FS: axotomy followed by coaptation with fibrin sealant derived from snake venom; AX+CFS: axotomy followed by coaptation with commercial fibrin sealant.

Statistical differences between groups were only observed at four weeks after sciatic nerve coaptation. In this sense, AX+FS displayed better recovery as compared to AX+CFS. Such statistical evaluation is presented in detail as supplementary material: Figure S4, fiber diameter; Figure S5, axon diameter; and Figure S6, myelin thickness. No statistical differences were depicted regarding the “*g*” ratio (Figure 9), although CFS group presented mostly under myelinated fibers four weeks after injury.

### 4.3. Spinal Cord Ultrastructural Changes following Nerve Coaptation with Fibrin Sealant

**Figure 10:**
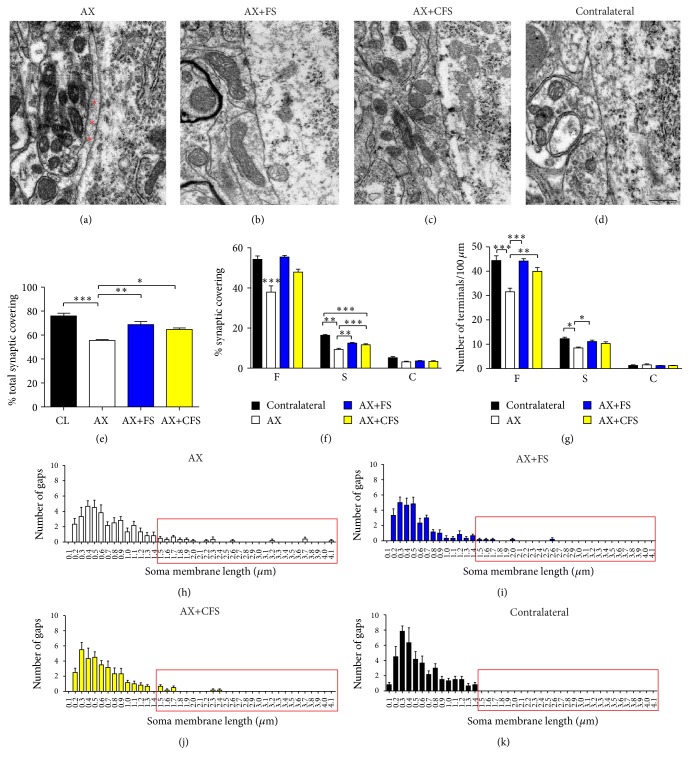
Representative ultrastructure micrographs showing synapses opposed to *α*-motoneurons, following P2 sciatic nerve transection and repair. (a) Terminal intermingled with an astrocyte projection (^*∗*^glial cell), following axotomy without repair. (b, c) Observe a close to normal synaptic apposition in neurorrhaphy groups, AX+FS and AX+CFS, respectively. (d) Contralateral side. Scale bar = 500 nm. (e) Quantitative ultrastructural analysis of synaptic covering. (f) Detailed quantitative analysis of F, S, and C terminals. (g) Synaptic covering with normalized number of boutons per 100 *μ*m of motoneuron membrane. Observe a reduction in synaptic covering following axotomy alone, 4 weeks after lesion. (h, i, j, k) Distribution of gap length between clusters of terminals apposing to motoneuron membrane in all groups analyzed. Observe that normal neurons (k) present presynaptic terminals close to each other, not exceeding 1.4 *μ*m gap. Nerve lesion without repair results in longer gaps between terminals (h) and nerve coaptation reduced such distances (i, j; red rectangles). AX: axotomy; AX+FS: axotomy followed by neurorrhaphy with fibrin sealant derived from snake venom; AX+CFS: axotomy followed by neurorrhaphy with commercial fibrin sealant. Mean ± SE.

All analyzed motoneurons showed at least one cholinergic presynaptic terminal (type C) in apposition to the plasmatic membrane surface. Lesioned neurons, affected by axotomy, showed changes compatible with chromatolysis, such as displacement of the nucleus to the periphery and decrease in cytoplasm electron density. Glial projections were observed, in the AX group, intermingling the space between part of the presynaptic terminals and the postsynaptic membrane (Figure 10(a)). Fully apposed terminals were depicted in the other experimental groups (Figures 10(b)–10(d)). The total synaptic covering, which represents the motoneuron body surface in contact with presynaptic terminals, revealed reduction of excitatory terminal covering in all groups analyzed, when compared to the contralateral side (Figure 10(e)).

Four weeks after lesion, nontreated neurotmesis (AX group), presented a 41.22% covering reduction of type S inputs when compared with contralateral side. Nerve coaptation resulted in excitatory input preservation, with no statistical differences between commercial and CEVAP sealant (Figures 10(f) and 10(g)). Fibrin sealant nerve repair promoted even more prominent effects on inhibitory inputs (type F boutons), showing close to normal covering (Figures 10(f) and 10(g)).

The gaps between clusters of terminals in apposition to the postsynaptic membrane were measured. Axotomy alone led to greater distance between terminals that contrasted with coaptation repair, where clusters of terminals were close to each other (Figures 10(h)–10(k)).

### 4.4. Nerve Coaptation Results in Significant Partial Recovery of the Peroneal Index as well as in the Nociception Recovery

**Figure 11:**
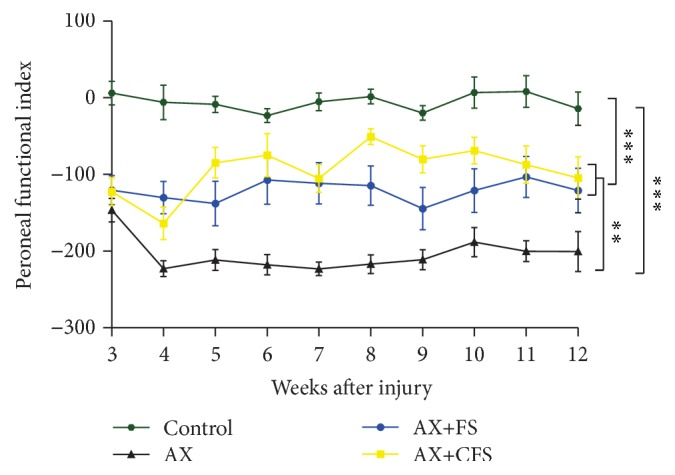
Motor function recovery up to 12 weeks after lesion by the analysis of the peroneal nerve functional index. Observe the significantly better performance of coaptation groups (AX+FS and AX+CFS) when compared with axotomy without repair group (AX). AX: axotomy; AX+FS: axotomy followed by coaptation with fibrin sealant derived from snake venom; AX+CFS: axotomy followed by coaptation with commercial fibrin sealant; control: unlesioned group.

The recovery of motor function was studied by the walking track test, using the CatWalk System (Noldus Inc.). Statistical analysis from peroneal functional index showed a significantly greater peroneal functional index (PFI) following coaptation (AX+FS, AX+CFS), regardless the nature of the fibrin sealant (Figure 11). PFI has been chosen over sciatic functional index (SFI), because measuring the intermediate toe spread (ITS) was not possible. We regard that to muscle atrophy since nerve lesion was performed in the neonatal period. Nevertheless, PFI has successfully shown motor recovery, indicating axonal regrowth.

**Figure 12:**
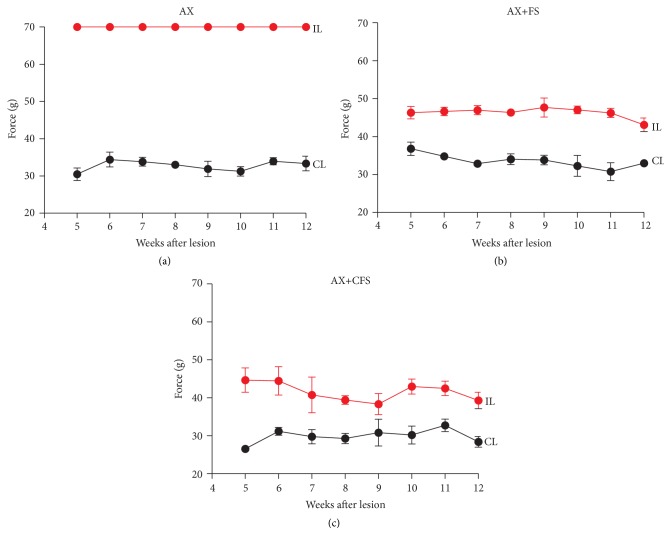
von Frey measurements (*n* = 5 per group, mean ± SE), 12 weeks after lesion, obtained from the right hind (CL, contralateral side, no lesion) and left hind paw (IL, ipsilateral-lesioned side). The values are expressed in grams (g) applied to trigger the “flinch” response. (a) Observe the complete absence of nociceptive response in the axotomy without repair group (AX). (b, c) Note significant nociceptive recovery of both coaptation groups (AX+FS and AX+CFS, resp.). AX: axotomy; AX+FS: axotomy followed by coaptation with fibrin sealant derived from snake venom; AX+CFS: axotomy followed by coaptation with commercial fibrin sealant.

The nociceptive recovery was analyzed by stimulation of the plantar surface of the paw with electronic von Frey. Contralateral paw showed withdraw reflex around 30 g. Axotomy alone (AX) rats did not show withdraw behavior at the cut-off weight, indicating lack of sensory perception (70 g, Figure 12(a)). Both groups subjected to coaptation with fibrin sealant (AX+FS and AX+CFS) showed lower threshold withdrawal response (around 45 g, Figures 12(b)-12(c)).

## 5. Discussion

It is well known that neonatal axotomy results in devastating neuronal loss in the spinal cord and dorsal root ganglia (DRG). This is particularly because of the interruption of neurotrophic factors production and retrograde flow, mostly at the target organs. In fact, administration of neurotrophic factors, such as brain derived neurotrophic factor (BDNF) and ciliary neurotrophic factor (CNTF) rescue motoneurons from degeneration [46]. Also, the use of cannabidiol (CBD), a cannabinoid with neuroprotective properties, is also able to avoid both motoneuron and DRG neurons death, following neonatal peripheral nerve lesion [47]. In this way, early neonatal nerve axotomy has been broadly accepted as a consistent model for studying neuroprotection [46, 48]. Nevertheless, due to the reduced size of pups, combined with the thin and delicate structure of the peripheral nerves at perinatal stage, repair following transection is hardly accomplishable.

The present work shows that end-to-end nerve coaptation can be performed in a reproducible fashion by using fibrin sealant as a connecting structure between proximal and distal stump. It is important to highlight that in humans nerve size is larger, allowing direct suturing. In this scenario, the use of fibrin glue may enhance repair stability and function as a scaffold for cell migration.

In the present study, we compared two different fibrin adhesives, one commercial brand and another recently developed by CEVAP. The latest is based on nonhuman components, avoiding transmission of infectious blood-borne diseases. The results have shown that both sealants (commercial and CEVAP) are excellent for the coaptation process, although CEVAP's fibrin sealant has proven to be easier to handle at the moment of surgery. Surgeries involving neonatal subjects ought to be particularly fast, and clotting speed is a crucial parameter. Due to the fact that CEVAP derived adhesive uses bubaline fibrinogen, the concentration of such protein in the cryoprecipitate is specially higher than the human counterpart [49]. In turn, coagulation threshold is significantly accelerated as well as the scaffold stability. This in turn possibly allowed early regrowth of regenerating axons, originated from the proximal stump, resulting in improved morphometric parameters, four weeks after repair. In line with the positive results described in the present work, the fibrin sealant from CEVAP has been tested in humans, for autologous skin grafting of the nasogenian sulcus showing promising results. Moreover, it has been tested for the immobilization of free periodontal gingival grafts in lower premolars and for the treatment of chronic venous ulcers [50–54]. Besides, recent studies also show the efficacy of this new sealant on peripheral nerve regeneration [55, 56], treatment of skull defects [57], and following ventral root avulsion [58].

Neuronal death is irreparable and results in sensibility and motor loss. It has been shown that this scenario can be partially reversed by local or systemic administration of trophic substances, such as neurotrophins, antioxidants, cannabinoids, and gangliosides [47, 59, 60]. Nevertheless, the regrowth of axons from the spinal motoneurons and DRG neurons towards the periphery is dependent on anatomical restauration of PNS structures. We show that coaptation at the neonatal stage is neuroprotective by itself and generates a permissive scaffold through which regeneration can be successfully accomplished. The results shown herein indicate significant preservation of motoneurons in the spinal cord, assessed by direct counting at 4, 8, and 12 weeks after lesion, reinforcing the long lasting positive effects of the repair procedure. Sensory and motor recovery is also present and could be evaluated by behavioral analysis. Nevertheless, since nerve repair was carried out at P2, motor and sensory evaluation could only be initiated from the third (CatWalk) or fourth (von Frey) week of age due to animal size and sensory-motor coordination. In turn, our data reflect already established recovery that happened before behavior tests could be started, what explains the rather constant peroneal and von Frey results.

Importantly, restauration of neonatal lesion, which mimics obstetric brachial plexus injury, led to preservation of synapses, as seen by synaptophysin immunostaining. Reduction of astrogliosis and microglial reaction also contribute to synapse stability and functionality, correlating with substantial motor and sensory recovery in AX+FS and AX+CFS groups up to twelve weeks after surgery. In this regard, improved peroneal index following coaptation was observed with either fibrin sealant treatment, when compared with axotomy alone. Such improvement indicates reestablishment of muscle innervation as well as suprasegmental control [61, 62]. Nociception reestablishment showed a similar time course, suggesting sensory axon regeneration [63].

After an injury, retraction of presynaptic boutons that appose spinal motoneurons takes place during the first week after lesion. This event reduces synaptic transmission [64–69] and allows the axotomized neurons to focus metabolism on survival and regrowth of a new axon and dendrites. Herein, synaptic covering was significantly preserved in coaptation groups, in line with the immunohistochemical observations (synaptophysin). A quantitative analysis of the synaptic covering for each type of presynaptic terminal showed significant preservation of S and F type of synapses. However, the proportion of preserved inhibitory synapses was greater than excitatory. Such scenario, according to Lindå et al. [70], facilitates neuroprotection by reducing glutamatergic excitotoxicity [70, 71]. Besides the prevention of excitotoxicity, the number of preserved presynaptic terminals/100 *μ*m in apposition to the alpha-motoneuron membrane was also increased in both coaptation groups.

Synapse dynamics also depend on astrocytes and microglia that act in favor of maintenance of homeostasis that is necessary to transmission and synaptic plasticity. One well-known retrograde effect of peripheral axotomy is the development of central astrogliosis and microglial reaction, directly interfering on synapse stability [72–75]. GFAP and Iba1 are highly expressed proteins in astrocytes and microglia, respectively, and are upregulated after lesion [73, 76]. The fact that coaptation downregulates glial reaction at lumbar spinal cord level is in line with the positive anatomical and behavioral findings previously discussed.

Overall, the present data suggest that acute repair of neonatal peripheral nerve with both fibrin sealant analyzed, namely, a commercial brand and a nonhuman derived adhesive produced by CEVAP/Brazil, promotes neuroprotection and regeneration of motor and sensory axons. Also, the present study demonstrates that neonatal end-to-end nerve coaptation is feasible and may in turn be of use for repairing obstetric brachial plexus injuries.

## Supplementary Material

Supplementary material represents additional experiments/illustrations provided to give additional support to the main results of the present work.

## Figures and Tables

**Table 1:** Regenerated sciatic nerve mean area (*µ*m^2^) following P2 sciatic nerve transection and repair. Mean ± SE.

Survival time (weeks after lesion)	AX+FS	AX+CFS	Contralateral
4	65,275 ± 12.66	43,281 ± 3.07	245,655 ± 20.12
8	68,791 ± 6.93	79,175 ± 6.95	419,924 ± 54.99
12	69,526 ± 6.43	92,425 ± 13.06	420,979 ± 20.77
