# Supplementary material for: Long-Standing Motor and Sensory Recovery following Acute Fibrin Sealant Based Neonatal Sciatic Nerve Repair
Source: Neural Plast. 2016 Jun 29;2016:9028126. doi: 10.1155/2016/9028126 (PMC4942656; doi:10.1155/2016/9028126)
Supplement: Supplementary file 1 — Supplementary material represents additional experiments/illustrations provided to give additional support to the main results of the present work. [file 9028126.f1.pdf]

Supplementary data

Table S1 - Percentage of motoneuron survival (ipsi/contralateral ratio) following P2 sciatic nerve transection and repair. Mean±SE.

Survival time (weeks post lesion)	AX	AX+FS	AX+CFS
**4**	20.42 ± 1.40	50.23 ± 3.68	49.40 ± 1.24
**8**	18.23 ± 2.67	43.64 ± 2.24	53.12 ± 3.72
**12**	16.81 ± 1.47	47.43 ± 1.34	48.93 ± 3.39

Table S2 – Synaptophysin relative immunoreactivity (ipsi/contralateral ratio) obtained by measurement of the integrated density of pixels at lamina IX ventral horn spinal cord, following P2 sciatic nerve transection and repair. Mean±SE.

Survival time (weeks post lesion)	AX	AX+FS	AX+CFS
**4**	0.25±0.02	0.61±0.05	0.95±0.01
**8**	0.41±0.03	0.71±0.05	0.87±0.03
**12**	0.35±0.01	0.79±0.05	0.88±0.03

Table S3 – Glial fibrillary acidic protein (GFAP) relative immunoreactivity (ipsi/contralateral ratio) obtained by measurement of the integrated density of pixels at lamina IX ventral horn spinal cord, following P2 sciatic nerve transection and repair. Mean±SE.

Survival time (weeks post lesion)	AX	AX+FS	AX+CFS
**4**	2.12±0.18	1.67±0.12	1.58±0.15
**8**	1.78±0.20	1.30±0.10	1.31±0.08

**12**	1.62±0.08	1.37±0.10	1.51±0.15

Table S4 – Ionized calcium-binding adapter molecule 1 (Iba1) relative immunoreactivity (ipsi/contralateral ratio) obtained by measurement of the integrated density of pixels at lamina IX ventral horn spinal cord, following P2 sciatic nerve transection and repair. Mean±SE.

Survival time (weeks post lesion)	AX	AX+FS	AX+CFS
**4**	1.49±0.06	1.07±0.05	1.07±0.03
**8**	1.21±0.09	1.14±0.05	1.19±0.08
**12**	0.90±0.27	1.24±0.04	1.15±0.05

Table S5 - Estimated number of regenerated myelinated axons following P2 sciatic nerve transection and repair. Mean±SE.

Survival time (weeks post lesion)	AX+FS	AX+CFS	Contralateral
**4**	2,129±579	2,881±304	6,776±183
**8**	1,999±235	2,508±590	8,131±437
**12**	2,119±284	3,156±510	8,458±386

Supplementary Figures

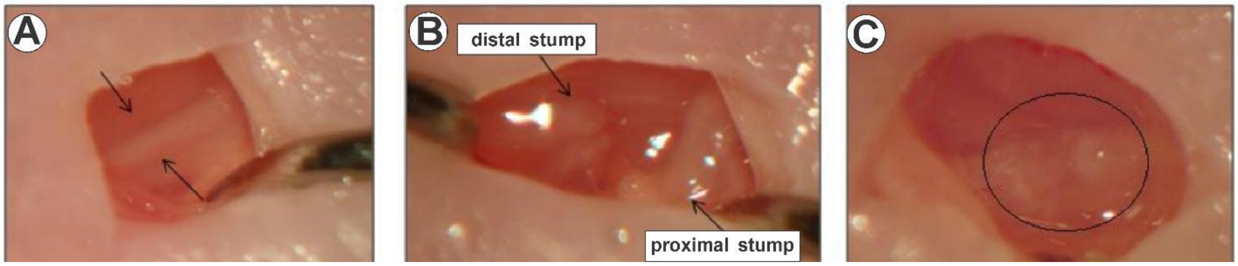

Figure S1. Neonatal sciatic nerve repair technique, by stump reapposition and application of fibrin sealant. **A.** Arrows indicate intact sciatic nerve previously to transection. **B.** transected sciatic nerve. Arrows indicate proximal and distal stumps previously to repair; **C.** nerve coaptation with fibrin sealant. Circle indicates fibrin matrix connecting the stumps.

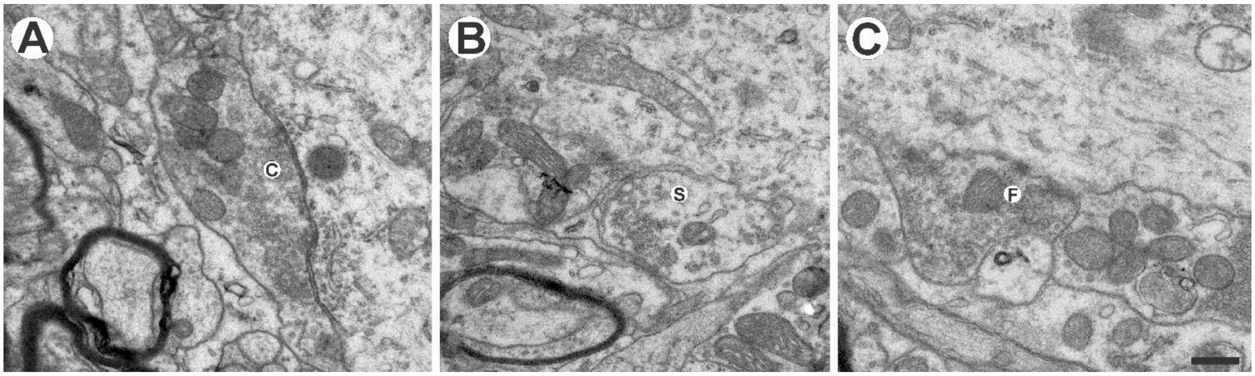

Figure S2. Representative transmission electron micrographs of S-, C-, and F-type terminals. **A.** Cholinergic bouton (C-type). **B.** Glutamatergic bouton (S-type). **C.** Glycine/GABA bouton (F-type). Scale: 500nm.

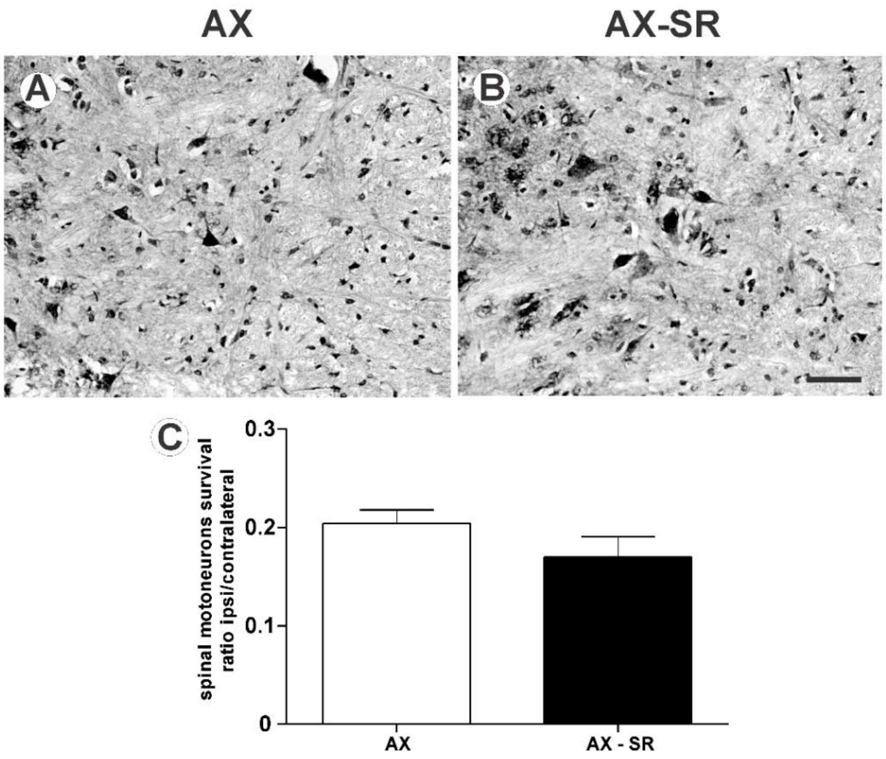

Figure S3. Nissl-stained spinal cord transverse sections at lamina IX illustrating the effect on motoneurons 4 weeks following P2 sciatic nerve transection. **A.** Group AX (axotomy, which a 2mm segment of the proximal stump was resected); **B.** Group AX-SR (axotomy, following stump reapposition); **C.** Ratio of neuronal survival four weeks following P2 sciatic nerve transection. Note that there is no difference between both groups. Mean±SE.

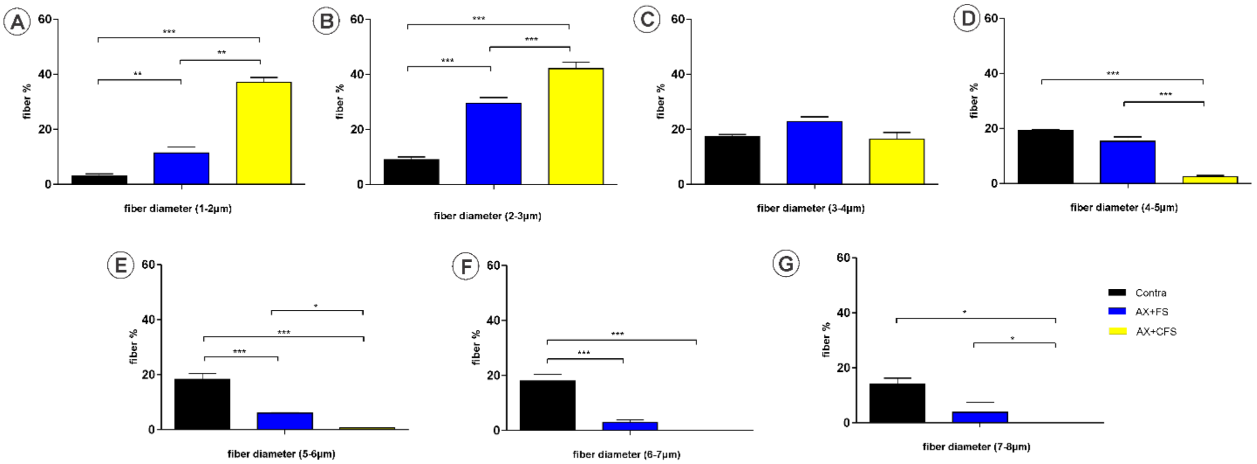

Figure S4. Quantitative analysis of regenerated fibers diameter, 4 weeks following P2 sciatic nerve transection and repair. Statistical differences between groups among frequency intervals (1-8µm). Note larger fiber diameter in the group AX+FS as compared to AX+CFS. Contralateral- non-lesioned sciatic nerve; AX+FS - axotomy followed by coaptation with fibrin sealant derived from snake venom; AX+CFS - axotomy followed by coaptation with commercial fibrin sealant. Mean±SE.

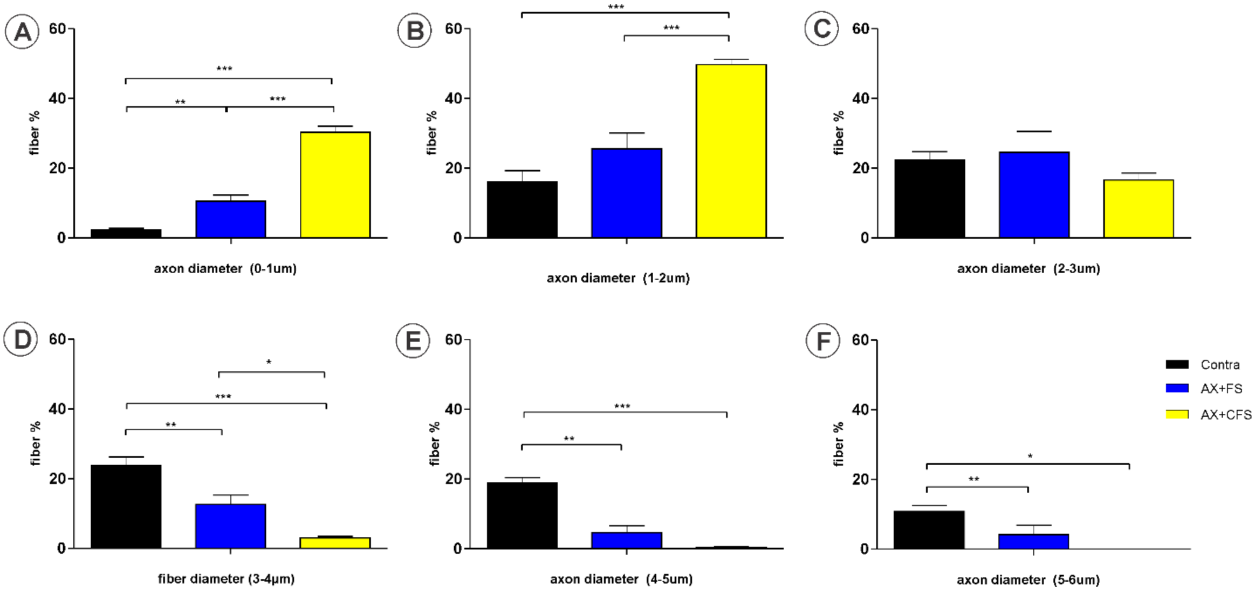

Figure S5. Quantitative analysis of regenerated axon diameter of, 4 weeks following P2 sciatic nerve transection and repair. Statistical differences between groups among specific diameters (0-6µm). Note the presence of larger fibers in the group AX+FS as compared to the group AX+CFS. Contralateral - non-lesioned sciatic nerve; AX+FS - axotomy followed by coaptation with fibrin sealant derived from snake venom; AX+CFS - axotomy followed by coaptation with commercial fibrin sealant. Mean±SE.

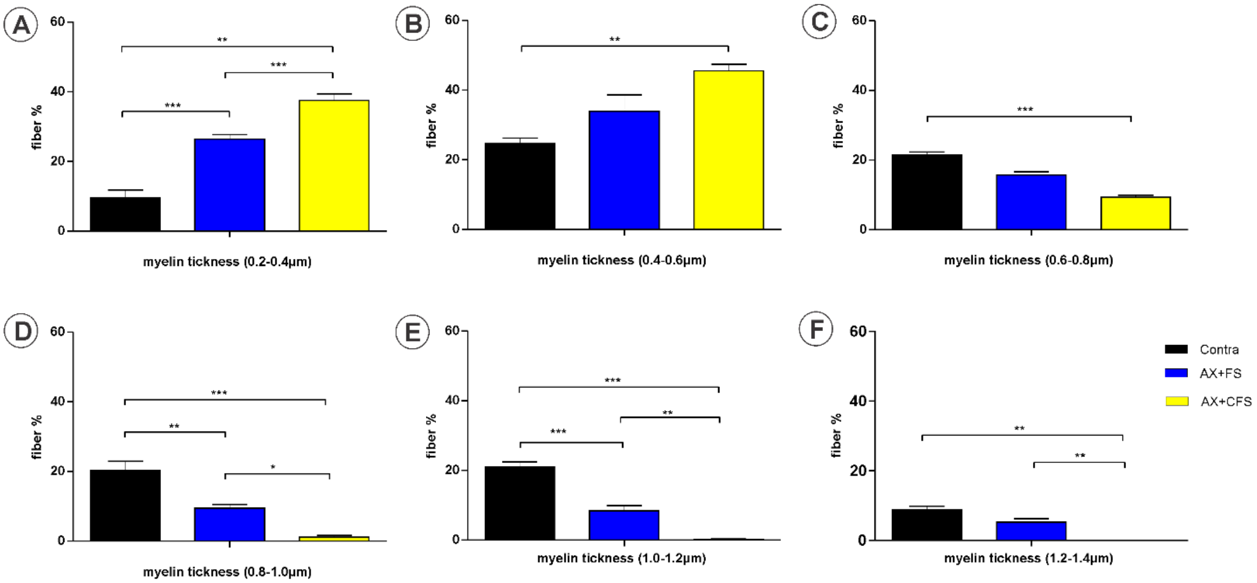

Figure S6. Quantitative analysis of myelin thickness of regenerated fibers, 4 weeks following P2 sciatic nerve transection and repair. Statistical differences between groups in specific thickness frequency intervals (0.2-1.4µm). Note increase of larger myelin thickness in the group AX+FS than in the group AX+CFS. Contralateral - non-lesioned sciatic nerve; AX+FS - axotomy followed by coaptation with fibrin sealant derived from snake venom; AX+CFS - axotomy followed by coaptation with commercial fibrin sealant. Mean±SE.
